# Supplementary material for: Agentic Discovery of Non-Canonical Antimicrobial Peptides with AMPGAN v3
Source: ArXiv. 2026 Jun 15:arXiv:2606.17127v1. Preprint. [Version 1] (PMC13308455)
Supplement: Supplement 1 [file NIHPP2606.17127v1-supplement-1.pdf]

A. In Vitro Experimental Protocol

Selected peptide candidates were commercially synthesized by a MedChemExpress(MCE) using standard solid-phase peptide synthesis and purified by high-performance liquid chromatography (HPLC). Peptide identity and purity were confirmed by mass spectrometry and analytical HPLC according to the quality-control reports.

Table 7. Bacterial isolates used in the current study.

Bacteria	Strain ID	Strain Designation	Source
S. aureus	USA300-0114	NRD384, MRSA	Wound, Mississippi, USA
B. subtilis	—	—	—
S. epidermidis	ATCC 35984	NRS 101	Catheter sepsis, Tennessee, USA
E. faecium	HM-952	503	Human, USA
E. coli	ATCC 25922	Seattle 1946	Washington, USA

B. AMPGAN v3: Implementation Details and Additional Analyses

All training was conducted on an NVIDIA RTX PRO 6000 Blackwell GPU, and all inference was performed on an NVIDIA GeForce RTX 4080.

B.1. Note on D- vs L-Amino Acids in Evaluation

A central contribution of AMPGAN v3 is the ability to generate sequences containing non-canonical residues (D-amino acids) and N/C-terminus modifications. We emphasize a distinction between two settings in this paper:

- **In vitro evaluation (Section 5.1).** Candidates synthesized for wet-lab testing were drawn from the *full* generative vocabulary, including D-amino acid substitutions and C-terminal amidation, since these modifications are indispensable for therapeutic viability (Lucana et al., 2021). This setting reflects the intended use case of AMPGAN v3.
- **In silico evaluation (Section 5.2 and Tables 4–5).** For all quantitative comparisons against AMPGAN v2, HydrAMP, and AMP-Designer, we generate sequences using the *L-amino acid subset* of our vocabulary only. None of the baseline models support D-amino acids or terminal modifications, and the external classifiers (AMP Scanner v2, amPEP) and embedding models (ESM2) used for evaluation are trained exclusively on canonical L-peptides. Including non-canonical residues in the comparison would have introduced an out-of-distribution artifact that confounds rather than informs the comparison. Restricting to L-only generations ensures the gains we report reflect modeling improvements rather than vocabulary differences.

B.2. Hyperparameters and Training Configuration

Table 8. AMPGAN v3 training hyperparameters.

**Generator**		**Discriminators**	
Latent dim d_z	256	d_{model}	64
Condition embed d_e	128	FFN hidden d_{hid}	256
Transformer L / H	4 / 4	Transformer L / H	4 / 4
Hidden dim	128	MLP head dim	64
Gumbel temp τ	1.0	Dropout	0.2
**Loss weights**		**Optimization**	
λ_ℓ (length)	0.5	Optimizer	AdamW, β=$(0.5, 0.999)$, wd 10^{-4}
λ_s (similarity)	0.5	LR (G / D_{adv} / D_{mic})	10^{-4} / 5×10^{-6} / 10^{-4}
λ_a (adversarial)	2	Batch size	256
λ_m (MIC)	1	Epochs	200
α (padding)	0.1	D : G updates	1 : 1

B.3. Token Vocabulary

Each sequence is encoded as `[N-term][SOS][Amino Acid Tokens][EOS][C-term]`, with maximum length $L = 64$ residues. The full vocabulary (Table 9) contains $V = 48$ tokens spanning canonical L-amino acids, D-amino acid stereoisomers, sequence-boundary markers, padding, and N/C-terminus chemical modifications. Lowercase letters denote D-amino acids, preserving stereochemistry directly in the token stream. This unified representation allows AMPGAN v3 to jointly generate sequence content and chemical modifications within a single output stream, in contrast to prior generative AMP models that operate over the 20 canonical L-amino acids only.

Table 9. AMPGAN v3 token vocabulary. Lowercase tokens denote D-amino acid stereoisomers of the corresponding uppercase L-amino acid. `<C8>`–`<C16>` denote fatty-acid acylation at the N-terminus with the indicated chain length.

Category	Tokens
L-amino acids (20)	A, C, D, E, F, G, H, I, K, L, M, N, P, Q, R, S, T, V, W, Y
D-amino acids (16)	a, c, f, h, i, k, l, n, p, q, r, s, t, v, w, y
Sequence boundaries	<SOS>, <EOS>
Sequence padding	<blank>
N-terminus modifications	<ACT> (acetylation), <C8>, <C10>, <C12>, <C14>, <C16>, <nblank>
C-terminus modifications	<AMD> (amidation), <cblank>

C. Agentic Pipeline: Architecture, Prompts, and Trajectories

C.1. System Prompts

Planning Agent.

```
You are a **Planning Agent** orchestrating a multi-agent system for
**Antimicrobial Peptide (AMP) Discovery**.
Your role is to coordinate the workflow by selecting the next agent and issuing
structured instructions in strict JSON format.
### Available Agents
{Agent Description}
{Agent Context} ---
### Planning Logic - If no peptide sequences exist, you MUST generate sequences
first.  - If peptide sequences exist but are unfiltered, you MUST filter.  - If
filtered results satisfy all constraints, you MUST select **END**.  - You MAY
iterate between Agents to refine results.
---
### Output Rules (STRICT) - Output ONLY JSON - No explanations, no extra text -
Must follow schema exactly - Do NOT assume hidden state | rely only on explicitly
provided input context
---
### JSON Format
{
  "Planning":  {
    "Agent":  "Generating | Filtering | Verifying | END",
    "Purpose":  "2--3 sentence description of the current objective for the
selected agent.",
    "Context":  {
      "instructions":  "Provide clear, structured instructions for the selected
agent to complete the assigned task.  This should describe what to do, not raw
input data.",
      "details":  "Contains all execution-critical information such as
user-provided inputs, parameters, constraints, tool-call arguments, or any
supplemental data required for the agent to perform its function accurately."
    }
  }
}
```

Execution Agent.

```
You are a **{Agent} Agent** designed to execute tool-based biological workflows for
**Antimicrobial Peptide (AMP)** tasks.  Your role is to follow the instructions
provided by the **Planning Agent** (your supervisor) and perform concrete actions
using appropriate biological tools.

### Context - **Planning Agent Instruction:**

{Instruction}

- **Your Capabilities:**

{Agent Description}

### Rules 1.  Output ONLY valid JSON matching the schema below | no prose, no
explanation.  2.  Each step is exactly one tool invocation.  Steps are numbered
sequentially from 1.  3.  Use only tools listed in your Agent Description.  All
required parameters must be provided.  4.  You may call the same tool multiple
times if needed.  5.  You may add calls to reporting/support tools from your
description if they improve output quality, but never invent tools.

### Output Schema

{
  "Agent":  "{Agent}",
  "Objective":  "<concise task summary>",
  "Steps":  [
    {
      "id":  1,
      "Tool":  "<tool_name>",
      "Input":  { "<param>":  "<value>" }
    }
  ]
}
```

C.2. Example Outputs

Planning Agent.

```
{
  "Planning":  {
    "Agent":  "Generating",
    "Purpose":  "Generate the initial batch of Antimicrobial Peptide
sequences targeting E. coli using AMPGAN-v3.",
    "Context":  {
      "instructions":  "Use the AMPGAN_v3 tool to generate an initial batch
of sequences targeting E. coli.  Generate exactly 5 sequences for the
first pass, with lengths between 10 and 20 amino acids.",
      "details":  {
        "min_length":  10,
        "max_length":  20,
        "num_generations":  5,
        "species_of_interest":  "ecoli",
        "folder_path":  "PATH"
      }
    }
  }
}
```

Execution Agent.


```
{
  "Agent": "Verifying",
  "Objective": "Cross-reference filtered AMP candidates against
SwissProt and DBAASP databases to assess novelty and retrieve biological
annotations.",
  "Steps": [
    {
      "id": 1,
      "Tool": "Verify_SwissProt",
      "Input": {
        "folder_path": "PATH"
      }
    },
    {
      "id": 2,
      "Tool": "Verify_DBAASP",
      "Input": {
        "folder_path": "PATH"
      }
    }
  ]
}
```


C.3. Executor Report Example

Execution Overview The objective of this workflow was to refine a set of generated antimicrobial peptides (AMPs) by applying a sequence of four specific filters: length, cationicity, hydrophobicity, and D-amino acid composition. These filters were applied to a dataset located at "PATH" to identify high-quality candidates.

Step-wise Results The length filter was applied to select sequences between 10 and 20 amino acids, followed by a cationicity filter (2.0 - 8.0) and a hydrophobicity filter (-0.5 - 0.5). Finally, a D-amino acid filter was executed. For each of these four steps, the original CSV file was updated with corresponding indicator columns ('length_filter', 'cationicity_filter', 'hydrophobicity_filter', and 'damino_filter'), where a value of 1 marks a successful pass and 0 denotes a failure to meet the criteria.

Final Outcome The final status assessment confirms that, from the initial pool of 5 generated sequences, 1 sequence successfully passed all applied filters. This sequence met the requirements for length, cationicity, hydrophobicity, and D-amino acid composition, representing the single candidate remaining after the full filtering process.

Completed Tasks The successfully completed tasks include the Length Filter, Cationicity Filter, Hydrophobicity Filter, D-amino acid Filter, and the final Filter Status report.

C.4. Tool Specifications

Each executor agent operates over a fixed set of tools registered in its system prompt. Tools accept structured JSON inputs from the agent and output string-template reports describing their outcome (e.g., counts of surviving candidates, BLAST hits, generation summaries). The executor agent aggregates these per-tool reports into a single run report, which is routed back to

the Planning Agent as context for its next decision. This separation lets tools remain stateless and replaceable while the executor handles report composition. Below we list each agent's tools, their inputs, and operational notes that the Planning Agent uses when deciding whether to re-invoke.

Generating Agent. Translates user-defined natural-language constraints into structured tool inputs.

- `AMPGAN_v3` — GAN-based AMP generator (Section 3). Outputs are written to `generated_sequences.csv` for downstream tools. *Inputs*: `min_length`, `max_length` (int), `num_generations` (int), `species_of_interest` (str; one of `ecoli`, `paeruginosa`, `kpneumoniae`, `saureus`, `bsubtilis`, `sepidermidis`), `folder_path`.
- `Predict_Structure` — 3D structure prediction via SimpleFold (Wang et al., 2025d) 360M. Used only on filtered subsets to bound cost; the Planning Agent is instructed to defer this tool until after physicochemical filtering. *Inputs*: `folder_path`.

The Generating Agent follows an adaptive batch-sizing policy: the first invocation generates the user-requested count N; after observing the post-filter pass rate p, subsequent rounds generate $\lceil S/p \rceil$ candidates to cover the shortfall S, capped at $3N$ per call.

Filtering Agent. Applies sequential physicochemical and structural screens. Each filter writes a boolean column to the candidate CSV and the Filter_Status tool reports the surviving count. The Planning Agent prioritizes cheap property filters before invoking structure-dependent filters.

- `Cation_Filter` — Filters by net charge at pH 7. *Inputs*: `min_cationicity` (float), `max_cationicity` (float), `folder_path`.
- `Hydrophobicity_Filter` — Filters by GRAVY score, balancing membrane insertion against aqueous solubility. *Inputs*: `min_hydrophobicity` (float), `max_hydrophobicity` (float), `folder_path`.
- `Length_Filter` — Filters by amino acid count. *Inputs*: `min_length` (int), `max_length` (int), `folder_path`.
- `Structure_Filter` — Classifies predicted PDB structures into one of six classes: mixed, α-helical, β-hairpin, extended β-strand, structured turns, or unstructured. Requires PDB output from `Predict_Structure`; does not perform folding itself. The `structure` input is a semicolon-separated string of class IDs(e.g., `"2;3"`). *Inputs*: `folder_path`, `structure` (str).
- `Damino_Filter` — Retains sequences containing at least one D-amino acid (lowercase letter in the token stream). Invoked when the user objective specifies non-canonical chemistry. *Inputs*: `folder_path`.
- `Filter_Status` — Mandatory bookkeeping tool. Reports the integer count of surviving candidates after each filtering cycle. The Planning Agent uses this output to decide between proceeding, re-filtering, and re-generating.*Inputs*: `folder_path`.

Verifying Agent. Cross-references filtered candidates against external databases to assess novelty and biological context. Both BLAST searches return the top hit per candidate within an E-value threshold of 10. While elevated E-value cutoffs are standard for short-peptide BLAST searches, the threshold here is set permissively for demonstration and is exposed as a tunable parameter.

- `Verify_SwissProt` — Local BLAST (Camacho et al., 2009) alignment against SwissProt (Boutet et al., 2007), returning top homologs with taxonomic classifications and cross-referenced annotations. *Inputs*: `folder_path`.
- `Verify_DBAASP` — BLAST alignment against the DBAASP (Pirtskhalava et al., 2020) subset used to train AMPGAN v3, returning the closest training-set match together with reported antimicrobial activity. Used to assess training-set proximity and recover known-activity annotations for near-duplicates. *Inputs*: `folder_path`.

C.5. Representative Trajectory

We include a representative trajectory from a multi-agent run with objective "Generate 5 AMPs with D amino acid targeting *E. coli* with α-helical secondary structure."

1 **Planning Agent → Generating Agent**
Execute AMPGAN_v3 to generate a batch of AMP sequences based on the user's initial request. Ensure length constraints and target species are applied.
# of Generated: 5
# of Total Generated: 5

2 **Planning Agent → Filtering Agent**
Execute the length, cationicity, hydrophobicity, and D-amino acid filters on the generated sequences. Once all preliminary filters are applied, use the Filter_Status tool to report the current count of surviving candidates.
# of Filtered: 1

3 **Planning Agent → Generating Agent**
Use AMPGAN_v3 to generate 20 more peptide sequences targeting E. coli. This calculation is based on needing 4 more candidates and observing a 20% pass rate in the initial filtering round.
# of Generated: 20
# of Total Generated: 25

4 **Planning Agent → Filtering Agent**
Run Damino_Filter to ensure D-amino acids are present, followed by Length_Filter, Cation_Filter, and Hydrophobicity_Filter. Conclude with Filter_Status to report the number of surviving candidates.
# of Filtered: 3

5 **Planning Agent → Generating Agent**
Run AMPGAN_v3 to generate a second batch of sequences. Based on the previous pass rate (3 out of 25 = 12%), generate 17 additional sequences targeting E. coli to compensate for the shortfall of 2 candidates.
# of Generated: 17
# of Total Generated: 42

6 **Planning Agent → Filtering Agent**
Execute the fundamental property checks on the updated sequence pool. Apply the length filter, cationicity filter, hydrophobicity filter, and D-amino acid filter. Finally, invoke the Filter_Status tool to report the total count of candidates that have survived these preliminary screens.
# of Filtered: 3

7 **Planning Agent → Generating Agent**
Use AMPGAN_v3 to generate a supplemental batch of sequences targeting Ecoli. Calculate the number of sequences to generate based on the shortfall and observed pass rate, but strictly cap this generation call at 3 times the requested count (15 sequences) to bound costs.
# of Generated: 15
# of Total Generated: 57

8 **Planning Agent → Filtering Agent**
Execute the fundamental property checks on the updated dataset. Apply the length, cationicity, hydrophobicity, and D-amino acid filters to isolate sequences meeting the user's constraints. Conclude by calling the Filter_Status tool to report the surviving candidate count.
# of Filtered: 5

9 **Planning Agent → Generating Agent**
Execute the Predict_Structure tool on the refined subset of 5 sequences to generate PDB files. This step is strictly required before the structural classification filter can isolate alpha-helical conformations.

10 **Planning Agent → Filtering Agent**
Execute the Structure_Filter tool to classify and select peptides that form an alpha-helical conformation based on the recently predicted 3D structures. Afterward, run the Filter_Status tool to report the current count of surviving candidates.
# of Filtered: 5

11 **Planning Agent → Verifying Agent**
Execute the Verify_SwissProt tool to cross-reference the 5 successfully filtered candidates against the protein database. Retrieve the closest matching sequences, their taxonomic classifications, and relevant annotations.
